# Development of nanoemulsion formulation loaded with *Enteromorpha intestinalis* extract: Characterization and evaluation for topical use

**DOI:** 10.1371/journal.pone.0343626

**Published:** 2026-03-06

**Authors:** Shumaila Qadir, Shahlla Imam, Syed Abid Ali, Fatima Ramzan Ali, Wajiha Iffat, Rabia Ismail Yousuf, Rafia Usman, Shahana Wahid, Iqbal Azhar

**Affiliations:** 1 Department of Pharmacognosy, Faculty of Pharmacy & Pharmaceutical Sciences, University of Karachi, Karachi, Sindh, Pakistan; 2 Department of Pharmacognosy, Institute of Pharmaceutical Sciences, Jinnah Sindh Medial University, Karachi, Sindh, Pakistan; 3 H.E.J. Research Institute of Chemistry, International Center for Chemical and Biological Sciences (ICCBS), University of Karachi, Karachi, Sindh, Pakistan; 4 Department of Pharmacy, SM Sohail University, Karachi, Pakistan; 5 Department of Pharmaceutics, Dow College of Pharmacy, Dow University of Health Sciences, Karachi, Sindh-Pakistan; 6 Department of Pharmaceutics, Faculty of Pharmacy & Pharmaceutical Sciences, University of Karachi, Karachi, Sindh-Pakistan; 7 Department of Chemistry, NED University of Engineering & Technology, Karachi, Sindh, Pakistan; 8 Department of Pharmacy, Benazir Bhutto Shaheed University, Karachi, Sindh, Pakistan; Strides Pharma Science Limited, UNITED STATES MINOR OUTLYING ISLANDS

## Abstract

*Enteromorpha intestinalis* is a green seaweed enriched with diverse bioactive compounds that possess substantial pharmacological and biotechnological properties. Despite the focus on their therapeutic applications, research on the development of nano-formulations is limited. Therefore, this study aimed to develop an *E. intestinalis* (EI) extract-based nanoemulsion for topical use. Olive oil, Tween-80 (surfactant), and PEG-400 (co-surfactant) were selected for the formulation of the nanoemulsion. The Smix ratio was set to 1:1 using a pseudo-ternary phase diagram. Moreover, a design experiment ascertained the composition of the formulation, followed by physicochemical characterization. The optimal formulation, based on droplet size, was selected for further analysis. Stability studies, antioxidant and anti-inflammatory properties of selected nanoemulsion formulation were determined and acute dermal toxicity assay was also performed. The best formulation (F6) displayed a droplet size of 183.27 ± 20.04 nm, PDI of 0.6, and viscosity of 290 ± 5.77 m-Pa.S. The developed nanoemulsion exhibited good skin compatibility and a slightly acidic pH. Both the extract and nanoemulsion formulation exhibited concentration-dependent antioxidant activity. The nanoemulsion had a lower IC_50_ value of 163.19 μg/mL, showing greater efficacy than the seaweed extract alone. Formulation (F6) also significantly (p < 0.05) inhibited paw volume (8–31%) compared to the control, while diclofenac sodium achieved a maximum inhibition of 41%. The designed formulation was stable, effective, and non-irritating demonstrating its potential topical application. This study presents, for the first time, a nanoemulsion formulation that incorporates *E. intestinalis* extract. This advancement paves the way for further *in vivo* studies to assess the efficacy and safety of this formulation for clinical applications.

## Introduction

Nanotechnology is a cutting-edge scientific field that involves the development of materials and devices at the nanoscale, with tailored properties for diverse applications [1]. Nanomaterials, including nanomedicine, nano-pharmaceuticals, theranostics, and nano-imaging agents, have revolutionized disease diagnosis, prevention, and management strategies. Nanomedicine-based formulations offer several advantages, for instance, targeted drug delivery, enhanced efficacy, reduced toxicity, improved patient compliance, and favorable clinical outcomes, making them promising candidates for treating complex diseases like cancer. Their distinctive nanoscale dimensions and extensive surface area to volume ratio give more active sites, which facilitate precise interactions within the cells and tissues. To date, approximately 100 nanomedicine formulations have received approval from the European Medicines Agency (EMA) and the United States Food and Drug Administration (FDA), and the field is poised for further advancement. Nanomedicines, including Lipid-and polymer-based formulations, nano-crystals, inorganic nanoparticles, and protein-based nanoparticles, are currently commercially available [2]. In recent years, nano-formulations, particularly nanoemulsions (NEs), have gained considerable interest in various scientific fields. NEs are classified into four major categories: O/W (Oil-in- Water), W/O (Water-in-Oil), O/W/O (Oil-in-Water-in-Oil), and W/O/ W (Water-in-Oil-in-Water), based on the pattern of dispersion of the oil and water phase [3]. The small droplet size of nanoemulsions ranges from 20-500 nm, providing greater surface area, long-term stability, better penetration, and enhance biological activity of the bioactive compounds. Therefore, finds applications in various domains, such as pharmaceuticals, cosmetics, personal care formulations, transdermal, vaccines, cell culture, cancer therapy, and in drug delivery systems [4–6].

Seaweed is a versatile natural resource that is gaining recognition as a promising material for developing safe, effective, and cost-efficient formulations [7]. Seaweed-based cosmetics have gained popularity as natural alternatives to synthetic products. The bioactive compounds in seaweed provide numerous skin care benefits, including antioxidant, antibacterial, and anti-aging properties [8]. Their diverse functional, sensorial, and biological properties make them ideal ingredients for developing innovative and effective cosmetic formulations. Many cosmetic companies are now incorporating seaweed extracts into their formulations as active agents, excipients, pigments, preservatives, additives, and fragrance agents. Seaweeds contain several bioactive compounds, primarily polysaccharides, along with polyphenolics, natural pigments, amino acids, proteins, peptides, polyunsaturated fatty acids, sterols, minerals, and vitamins. These bioactive compounds exhibit a wide range of bioactivities, making them suitable as active components in topical formulations. For instance, Gracilaria extracts are used in various commercial cosmetics, such as hydrogel soap, facial masks, and hydrating creams, while mycosporine-like amino acids are incorporated into in various sunscreen products. Seaweed-based ingredients are available in various forms including creams, hydrogels, soaps, shampoos, and sprays. Their effectiveness and stability can be enhanced using nanocarrier systems, such as nanoparticles, nanoemulsions, nanohydrogels, and liposomes, which facilitate the delivery of active agents for optimized effects [9,10].

Numerous seaweed species have been identified and characterized, yet several varieties widely disseminated along Karachi’s coastline remain underexplored. *Enteromorpha intestinalis*, formerly known as *Ulva intestinalis* (family: Ulvaceae), is one of the most abundant algal species found in coastal regions, such as Buleji, Korangi Creek, Manora, and Sandspit in Karachi, Pakistan [11]. *Enteromorpha intestinalis* is enriched with bioactive compounds, such as phenolics and flavonoids, which are potentially active against free radicals [12]. Though the extensive application of seaweed extracts in various fields has been established, their use in the development of nanoemulsions has been limited. Therefore, the present study aimed to develop a nanoemulsion formulation incorporating *Enteromorpha intestinalis* extract for topical delivery, which may offer potential benefits for both the cosmeceutical and pharmaceutical industries.

## Materials and methods

### Materials

Olive oil (Sasso, Italy) and virgin coconut oil were procured from a local supplier in Karachi, Pakistan. Ascorbic acid, DPPH (2, 2-diphenyl-1-picrylhydrazyl), ferric chloride, potassium ferricyanide, trichloroacetic acid, Tween 80, and PEG 400 were obtained from Sigma-Aldrich Co. (St. Louis, USA). While ethanol was purchased from Merck (Germany). A commercially available diclofenac sodium gel (2%) was obtained from a local pharmacy in Karachi, Pakistan. Analytical grade reagents and chemicals were utilized in this study.

### Seaweed material

*Enteromorpha intestinalis* was collected from the coastal area of Buleji, Karachi, Pakistan. Professor Dr. Mohtasheemul Hassan, from the Department of Pharmacognosy, Faculty of Pharmacy and Pharmaceutical Sciences, University of Karachi, Pakistan, authenticated the algal sample with the herbarium code EI-03–20.

### Preparation of *Enteromorpha intestinalis* extract

The seaweed was thoroughly washed several times with distilled water to eliminate impurities and unwanted debris. The sample was dried under shade and then ground into a fine powder. Approximately 500 g of powdered seaweed was macerated with ethanol (95%). The mixture was shaken manually every 12 hours for 7–10 days at room temperature [13]. After filtration, the algal material was concentrated in a rotary vacuum evaporator under reduced pressure. The algal extract was packed in a glass bottle and positioned in a refrigerator at 4 ^°^C for subsequent analysis.

### GC–MS (Gas chromatography–mass spectrometry) analysis of *E. intestinalis* extract

To identify the bioactive compounds in *E. intestinalis* extract, GC-MS analysis was performed as described by [12,14], with minor modifications to suit our specific sample matrix and equipment setup. For Chromatographic separation, a capillary column ZB-5 (30 m × 0.32 mm × 0.25 µm film thickness) and a SHIMADZU Nexis series GC-MS-TQ8040 NX System, including an auto sampler AOC-20i and a gas chromatograph GC-2030 coupled with triple Quad pole mass spectrometer, were used. Helium gas served as the carrier gas, with a flow rate of 1 mL/min, and a sample with a volume of 1 µL was injected. The oven temperature was initially set to 50 ^°^C for 5 min and then increased to 200 ^°^C at a rate of 5 ^°^C/ min over 10 min. Subsequently, the temperature rose to 300 ^°^C at a rate of 8 ^°^C/ min, which also lasted for 10 min. The total runtime for this process was 51 minutes. The temperature of the mass spectrometer’s transfer line was adjusted to 260 ^°^C, and the injector was maintained at 250 ^°^C. The solvent cut time before injection was 3 min. The Electron ionization (EI) system of the mass spectrometer was operated in full scan mode with an ionization energy of 70 eV, including fragments ranging from 20-600 *m/z*. The ion-source temperature was set to a constant value of 200 ^°^C. The GCMS real-time analysis software database of the National Institute of Standards and Technology (NIST) was used to identify the compounds.

## Nanoemulsion formulation

### Screening of nanoemulsion components

To determine the maximum solubility of the *E. intestinalis* extract in oil, two commonly used vegetable oils, virgin coconut oil and olive oil, were evaluated [15–17]. A variable amount of EI extract was added to 5 mL of the oil, followed by sonication (E 30H, Elmasonic, Germany) for 15 minutes at 37 ^º^C. The resultant mixture was then homogenized for an additional hour on a magnetic stirrer. Subsequently, the mixture was thoroughly mixed utilizing a vortex mixer (Whirl Mixer Lab, FSA Supplier, England) for 5 minutes, followed by centrifugation (Heraeus Biofuge Primo R Centrifuge, Thermo Electron Corporation) at 4000 rpm for 15 minutes. After that, the solution was visually observed to identify the residues [16,18]. The entire procedure was performed repeatedly, and the amount of extract in the oil was gradually increased (up to 1%, w/v), to identify the maximum concentration at which the oil could dissolve the extract. At 0.2% concentration, solubility increased significantly; however, exceeding this concentration resulted in a noticeable rise in residue formation, indicating that the solubilizing limit had been reached. The oil displaying the highest capacity to dissolve the algal extract was chosen [16]. Moreover, a UV spectrophotometer (Perkin Elmer SP-UV-500 Series UV-Vis Spectrophotometer) was utilized to evaluate the solubility of the extract in the aforementioned oils. The absorbance was noted at various wavelengths varied between 400 and 800 nm [17,19]. Tween 80 and PEG 400 were selected as surfactants and co-surfactants due to their low toxicity, biocompatibility, and extensive use in pharmaceutical formulations [3].

#### Pseudoternary phase diagram.

To delineate suitable formulation ranges, tween 80, PEG 400, and olive oil were chosen to construct a pseudo-ternary phase diagram utilizing the water titration method [20,21]. Various weight ratios of the surfactant and co-surfactant were mixed at 3:1, 2:1, and 1:1 to develop the optimal nanoemulsion formulations. Each Smix was then mixed with olive oil as the oil phase (weight ratio of 1:9–9:1). The oil and surfactant mixture was gradually diluted with distilled water (aqueous phase) while stirring constantly until equilibrium was achieved. The resultant sample was then observed visually to categorize as nanoemulsion, gel, or emulsion. The CHEMIX ternary plot software (Chemix School 9) was employed to construct the phase diagrams.

#### Formulation of *E. intestinalis* extract loaded-nanoemulsion.

According to the pseudo-ternary phase diagrams, the largest area (1:1 Smix) was selected for the formulation development. Furthermore, a Simplex lattice design experiment (Design Expert software Version 7.0) was employed to generate different compositions of nanoemulsion formulations [22]. In the low-energy method, spontaneous emulsification was used to prepare an *E. intestinalis* extract-loaded nanoemulsion. Under continual stirring, the aqueous phase was introduced drop wise into the oil phase, containing *E. intestinalis* extract (0.2%) and surfactant and co-surfactant mixture, previously homogenized for 5 minutes at room temperature utilizing a magnetic stirrer [21,23,24]. The resultant mixture was stirred for 1 hour until a clear, transparent, and equilibrium state (stable state) was attained. The NE formulation was set aside in a refrigerator at 4 ^°^C in a well-closed glass container till further use.

### Characterization of Nanoemulsions

**Droplet size and Polydispersity Index (PDI).** Particle size and polydispersity index (PDI) of the nanoemulsion formulations were determined utilizing the DLS (Dynamic Light Scattering) technique (Laser spectroscatter-201, Xtal Concepts, Germany). The analysis (n = 50) was executed at 25 ± 1 ^°^C with a fixed scattering angle of 90^°^. Before analysis, the sample was diluted 1:10 with deionized water to minimize the likelihood of light scattering. For the data analysis, Xtal Concepts software (Xtal Concepts, Germany) was utilized [25,26].

**pH determination.** The nanoemulsion formulation (1 g) was diluted with distilled water (10 mL). The pH of the resultant solution was noted at room temperature (25 ± 2 ^°^C) with a pH meter (Mettler MP-220, Schwerzenbach, Switzerland), [20]. Before the measurements, the pH meter was calibrated using standard buffer solutions with pH values of 4.0, 7.0, and 10.0 to ensure the accuracy and reproducibility of the obtained data [16]. Each measurement was performed in triplicate.

**Refractive index measurement.** The refractive index of the designed nanoemulsion was determined via an Abbe’s refractometer (Schmidt Haensch-24298, Germany). A small amount of the NE sample was introduced on the instrument’s glass slide, and the corresponding scale reading was recorded. Prior to the measurements, the instrument was initially calibrated with distilled water [27].

**Viscosity determination.** A Brookfield viscometer (Haake-19, Karlsruhe, Spain) equipped with an L3 spindle was utilized to assess the viscosity of the nanoemulsion. The spindle speed was set to 60 rpm. Triplicate measurements were taken at a constant temperature (25 ^**°**^C **±** 2) [28,29].

**Conductivity measurement.** The conductivity of the nanoemulsion formulation was measured at room temperature (25 ± 2 ^°^C) via a Conductometer (WPA-CMD-500, Cambridge, UK), [16,27].

### Model fitting and optimization

The developed nanoemulsion formulations were analyzed using a simplex lattice design experiment (Design Expert software Version7.0), with the aim to determine the effects of oil, Smix, and water percentages on droplet size, PDI, and viscosity. The design utilized the component ranges identified from the pseudo-ternary phase diagram: oil (5–15%), surfactant and co-surfactant mixture (Smix; 65–75%), and water (10–20%). A total of seven formulations were generated. Specific quantities of each ingredient were utilized to achieve a minimum droplet size, acceptable polydispersity index, and optimal viscosity. The independent variables were oil (X1), Smix (X2), and water (X3), while the dependent response variables were droplet size (Y1), polydispersity index (Y2), and viscosity (Y3). A quadratic model was employed for droplet size and PDI, whereas a linear model was used for viscosity. One-way ANOVA (analysis of variance) and the coefficient of determination (R^2^) were employed to evaluate the correlations between the independent and the response variables. A statistically significant *p*-value < 0.05 and a high R^2^ (>0.9) value indicate a strong, well-fitting model. Response surface curves were plotted to visualize the impact of these variables on the dependent responses [24,30]. Based on the response variables, the optimal formulation was chosen for further studies.

### Stability studies of nanoemulsion

Stability studies of the chosen nanoemulsion were performed at various time points, such as 0 months (fresh state), followed by 1, 2, and 3-months at 4 ± 2 ^°^C, and 25 ± 2 ^°^C. The physical appearance (phase separation, color change, clarity, and texture) and pH were evaluated. Furthermore, droplet size and polydispersity index were assessed at the fresh state, 48 hours, 15 days, and 3-month intervals [16,21,31].

### Extract-excipients interaction studies

Fourier Transform Infrared Spectroscopy (FT-IR) was employed to investigate the potential interactions between the components of the nanoemulsion and the extract. Approximately 20 mg of each sample (extract, olive oil, Tween 80, PEG 400, and nanoemulsion) was analyzed using an FT-IR Spectrophotometer (Nicolet Avatar 330; Thermo Electron Co., USA) equipped with a diamond attenuated total reflectance (ATR) accessory. The spectra were noted within a wave number range between 4000**−**400 cm ⁻ ¹ [17].

### Spreadability

The “Drag & Slip” method, with slight modifications, was employed for the evaluation of the spreadability of *E. intestinalis*-loaded nanoemulsion [32]. Two glass slides (each 7.5 cm long) were positioned parallel to one another. One slide was set as movable while the other one was in a fixed position. The fixed glass slide was connected to a pulley system, and around 1 g of nanoemulsion was placed between the slides. A 500 g weight was put on the slide for 5 minutes, followed by a 50 g weight connected to the movable side via a string that ran over the pulley. The time taken for the movable slide to be pulled off was noted. The below-mentioned equation was employed to determine the spreadability of nanoemulsion formulation, where S represents the spreadability of the designed formulation, M is the weight (g), while L indicates the length of the upper slide (cm), and T denotes the time (sec) taken to separate the two glass slides from each other.


S = M × L/T (1)

### Antioxidant activity

**DPPH free radical scavenging assay.** The free radical scavenging activity was evaluated using a slightly modified DPPH assay [28]. Approximately 2 ml of 2, 2-diphenyl-1-picrylhydrazyl (DPPH; 0.1 mM) solution mixed with 2 mL of varying concentration (50–800 µg/mL) of the *E. intestinalis* extract and nanoemulsion formulation (F6). Subsequently, the mixture was incubated in a dark chamber at room temperature for 20 minutes. The absorbance of each reaction mixture was then noted at 517 nm using a UV-Vis spectrophotometer. Ascorbic acid was used as a positive control (5–40 µg/mL). The equation indicated below was employed to determine the percentage inhibition, where Ac denotes the absorbance of the control and As represents the absorbance of the sample. Linear regression analysis was employed to determine the IC_50_ value.


Percentage inhibition = [(Ac−As)/ Ac× 100 (2)

**Ferric Reducing Power assay.** The ferric-reducing antioxidant power (FRAP) was performed as outlined in a previous study [33]. Aliquots of extract and nanoemulsion formulation (F6), ranging in concentration from 200–3200 µg/mL, were mixed with equal volumes (2.5 mL) of potassium ferricyanide (1%) and phosphate buffer (pH 6.6, 0.2 M). The resultant mixture was then incubated at 50 ^º^C for 20 minutes. Subsequently, 2.5 mL of 10% (w/v) trichloroacetic acid solution was introduced into the mixture, followed by centrifugation for 10 minutes. The supernatant (2.5 mL) was mixed with distilled water (2.5 mL) and 0.1% ferric chloride solution (0.5 mL). The absorbance was recorded at 700 nm against a blank solution. The results were presented as a percentage of 0.02 mg/mL of ascorbic acid, which served as a reference standard [34].

### Anti-inflammatory activity

**Animals.** Healthy male Albino Wistar rats (150–250 g) were obtained from the animal house of the Department of Pharmacology, Faculty of Pharmacy and Pharmaceutical Sciences, University of Karachi, Pakistan. The animals were housed in a controlled environment with a temperature of 22 ± 2 ^°^C, relative humidity of 50–60 ± 0.2%, and a 12- hour light/dark cycle. Standard diet and water were provided to the rats *ad libitum*. Before the study, the experimental animals were acclimatized to the laboratory conditions for a week. All experimental procedures adhered to the Good Laboratory Practice (GLP) guidelines and followed ethical protocols for animal care. The Institutional Bioethical Committee, University of Karachi, Karachi, Pakistan, approved this research study (approval No. IBCKU-141/2020/24).

**Carrageenan-Induced acute inflammation Model.** Anti-inflammatory activity was evaluated using a carrageenan-induced rat paw edema model, as previously described [35,36]. Animals were separated into three groups (n = 6). Group I served as the control (untreated), Group II was treated with a commercially available Diclofenac Sodium gel (2%), and Group III was treated with a nanoemulsion formulation. Before administering the carrageenan injection, the nanoemulsion formulation and diclofenac sodium gel (approximately 200 mg) were topically applied to the plantar surface of the right hind paw with gentle rubbing. Thirty minutes following the topical application, a carrageenan solution (0.1 mL, 1% w/v) was injected into the sub-plantar site of the right hind paw of each rat. Paw edema volume was measured at 30 minutes and 1, 2, 3, and 4 hours of post-carrageenan injection, using a plethysmometer (Ugo Basile 7140 Comerio, Italy) [37]. The initial paw volume (baseline) was noted for all animals before inducing inflammation. The percentage inhibition was determined using the equation given below, where Vt indicates the paw volume of the test group, and Vc denotes the paw volume of the control group [38].


% inhibition = 100 × (1 − Vt/Vc) (3)

### Acute dermal toxicity

An acute dermal irritation study was executed on Wistar Albino rats (150–250 g). The rats’ dorsal skin was shaved 24 hours prior to treatment application. Animals were separated into three groups (n = 6). Group I served as the control (untreated), while Group II received a commercially available 2% diclofenac gel. Group III was treated with algal extract-loaded nanoemulsion. Notably, all treatment groups (II and III) received approximately 200 mg of their respective formulations. After a 24-hour application period, the rats’ skin was examined for signs of rash, edema, and erythema using a 4-point severity scale (4; scar formation, 3; moderate redness/swelling, 2; well-defined redness/ swelling, 1; slight redness/swelling, and 0; no signs). The Primary irritability Index (PII) was calculated based on Draize dermal scores [17,39,40]. Data were analyzed to compare skin irritation between the experimental groups.

### Statistical analysis

The results were presented as mean ± SEM (Standard Error Mean). A Paired ‘t’-test (*P* < 0.05) was executed to assess the antioxidant activity. For the anti-inflammatory assay, one-way analysis of variance (ANOVA) followed by Dunnett’s post-hoc test (*P* < 0.05) was applied to determine the differences among the experimental groups. SPSS (Statistical Package for the Social Sciences, Inc., Corporation, Chicago, IL, USA) software version 27 was used for the data analysis.

## Results and discussion

### *E. intestinalis* extract and GC–MS analysis

**Fig 1:**
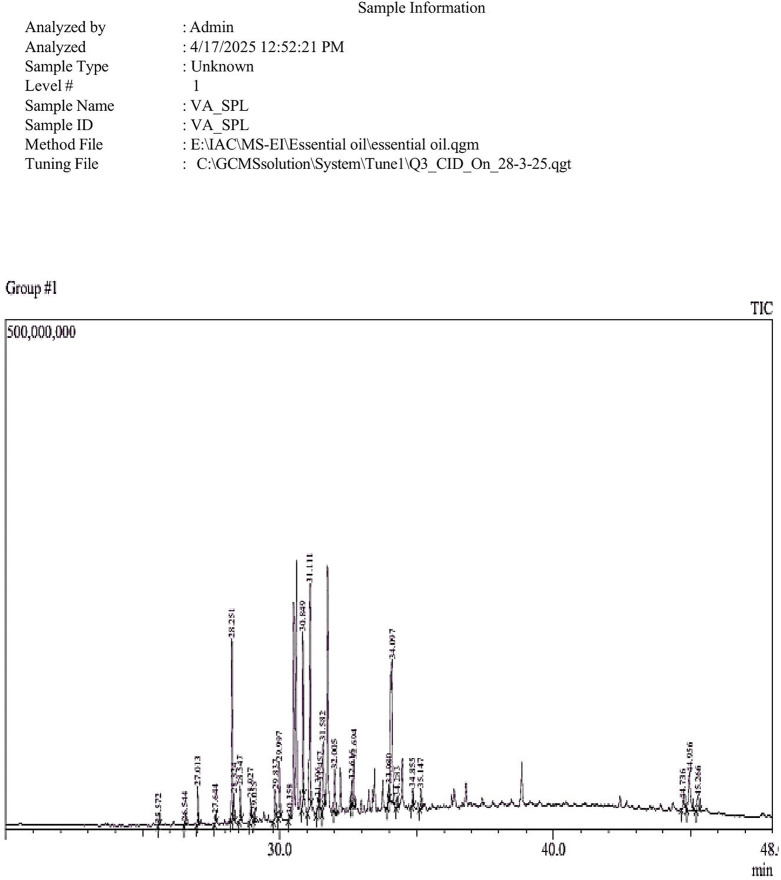
GCMS profile of *Enteromorpha intestinalis* extract.

The extraction of bioactive metabolites, including polyphenolics, carotenoids, flavonoids, saponins, minerals, and vitamins, poses several challenges owing to their complex nature. Several factors, such as solvent choice, temperature, pH, and extraction conditions, significantly influence the yield of crude extracts [41]. In this study, the yield of *E. intestinalis* extract was found to be 15.96%, which closely aligns with the previously reported yield of the ethanolic extract, documented as 13.4 ± 0.51% [42]. The GC–MS profile of *E. intestinalis* extract revealed a total of 28 major bioactive compounds (Table 1). These substances fall under different chemical classes, including hydrocarbons, hydrocarbon alcohols, phenols, sterols, terpenoids, and both saturated and unsaturated fatty acid esters. The majority of these compounds are said to have substantial biological activities. One of the key advantages of employing GC-MS is its exceptional precision in identifying derivatized compounds that are abundant in the sample [14]. The identified hydrocarbons were hexadecane, n-heptadecane, 1-heptacosene, n-nonadecane, neophytadiene, 2-methytetracosane, eicosane, and heneicosane. The fatty acid ester profile comprised saturated fatty acid esters, such as stearic acid, methyl ester; palmitic acid, ethyl ester; pentadecanoic acid, methyl ester, and tridecanoic acid, 12-methyl-, methyl ester, along with unsaturated fatty acid esters, including palmitoleic acid, methyl ester; oleic acid, methyl ester; elaidic acid, methyl ester; and oleic acid, eicosyl ester. The extract also contained phenol (2, 4- di-tert-butylphenol), alcohols (n-heptadecanol-1, n-nonadecanol-1, 3, 7, 11-Trimethyl-1-dodecanol), sterols (cholesta-4, 6-dien-3-ol, stigmasterol acetate), and derivatives of TMS (Isofucosterol, o-TMS). Additionally, terpenoids were present, including monoterpene lactone (Loliolide), diterpene alcohols (phytol, trans-phytol, 3, 7, 11, 15-Tetramethyl 1–2-hexadecen-1-ol), and a sesquiterpene ketone (hexahydrofarnesyl acetone). These findings are aligned with those of previous studies [12,14]. Notably, these metabolites are recognized for their diverse biological activities, including antioxidant, antimicrobial, cancer-preventive, and hyper-cholesterolemic properties [12]. The GCMS spectra of *E. intestinalis* extract are displayed in Fig 1.

**Table 1:** *E. Intestinalis* extract GCMS Peak Report TIC.

Peak#	R. Time	Area	Area %	Compound Name
1	25.572	2810229	0.08	2,4-Di-tert-butylphenol
2	26.544	7381695	0.21	Phytol
3	27.013	76449551	2.15	Hexadecane
4	27.644	16911841	0.47	3,7,11-Trimethyl-1-dodecanol
5	28.251	435243089	12.22	n-Heptadecanol-1
6	28.324	45264265	1.27	n-Nonadecanol-1
7	28.547	70118217	1.97	n-Heptadecane
8	28.927	52015733	1.46	Tridecanoic acid, 12-methyl-, methyl ester
9	29.055	17065166	0.48	1-Heptacosene
10	29.837	72069853	2.02	Loliolide
11	29.997	112996253	3.17	n-Nonadecane
12	30.358	11432887	0.32	Pentadecanoic acid, methyl ester
13	30.849	385536872	10.82	3,7,11,15-Tetramethyl-2-hexadecen-1-ol
14	31.111	689422525	19.35	Neophytadiene
15	31.396	29836499	0.84	2-Methyltetracosane
16	31.457	50077423	1.41	Palmitoleic acid, methyl ester
17	31.582	149950351	4.21	Hexahydrofarnesyl acetone
18	32.005	93432663	2.62	trans-Phytol
19	32.616	39769018	1.12	Palmitic acid, ethyl ester
20	32.694	100284836	2.82	Eicosane
21	33.980	46180906	1.30	Elaidic acid, methyl ester
22	34.097	689814803	19.36	Oleic acid, methyl ester
23	34.283	21146794	0.59	Stearic acid, methyl ester
24	34.855	38486687	1.08	Oleic acid, eicosyl ester
25	35.147	43279263	1.21	Heneicosane
26	44.736	33361975	0.94	Stigmasterol acetate
27	44.956	175423475	4.92	Cholesta-4,6-dien-3-ol
28	45.266	56443837	1.58	Isofucosterol, O-TMS
		3562206706	100.00	

### Screening of nanoemulsion components

**Fig 2:**
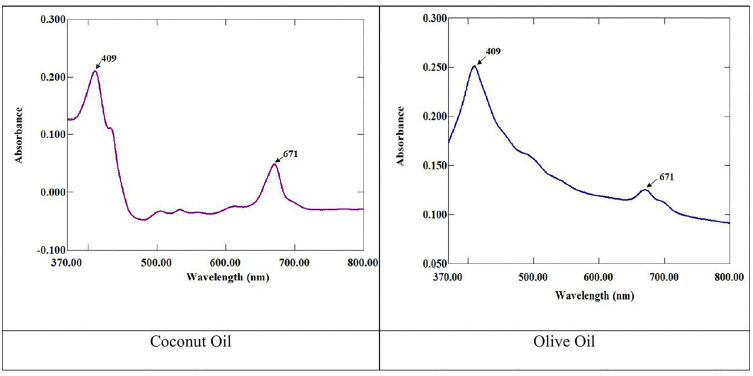
UV- spectral presentation of *E. intestinalis* extract (0.2%) in coconut and olive oil.

Nanotechnology has been extensively used in the pharmaceutical and cosmeceutical industries owing to its distinctive properties and exceptional ability to enhance the absorption of bioactive compounds. To achieve optimal nanoemulsions with desired properties, such as small droplet size, low polydispersity index (PDI), stability, and specific functionalities, various formulation factors are essential [43]. In the screening of nanoemulsion components, solubility studies were performed as a preliminary screening to identify the oil that could effectively solubilize the extract. Among the tested oils, olive oil demonstrated a higher solubilization potential for the extract than did virgin coconut oil. Furthermore, UV-Vis spectroscopy analysis substantiated these findings, displaying higher absorbance values in olive oil (Fig 2). An earlier study also employed UV-Vis spectrophotometry to characterize their formulation and reported a noticeable increase in absorbance, suggesting enhanced solubilization [20]. The greater solubility of olive oil is ascribed to its rich fatty acid composition, particularly monounsaturated fatty acids, like oleic acid, which have the potential to interact with the phytochemicals present in the extract [15]. Non-ionic surfactants are frequently chosen for nanoemulsion formulations owing to their reduced pH sensitivity, lower critical micelle concentration, and lower toxicity than ionic surfactants [23,44]. Consequently, Tween 80 and PEG 400 were selected as the surfactant and co-surfactant, respectively, owing to their promising physicochemical properties. This combination contributed to the emulsification process, yielding a clear nanoemulsion. A similar combination has been utilized in earlier studies [26,45,46]. PEG 400, Tween 80, and olive oil, employed in the designed formulation, have been successfully used in topical formulations, demonstrating their versatility and effectiveness [17,18].

### Pseudoternary phase diagram

**Fig 3:**
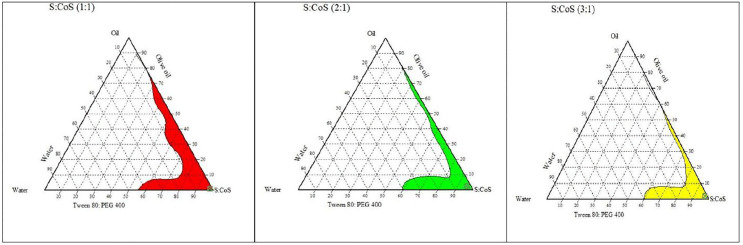
Pseudoternary phase diagram of olive oil with Smix. (Tween 80: PEG) and water at ratio of 1:1, 2:1, and 3:1.

Phase diagrams are an invaluable tool for comprehending the behaviour of nanoemulsions and predicting their stability based on component ratios [47]. Fig 3 shows the phase diagram of olive oil with Smix (Tween 80: PEG 400) and water at ratios of 1:1, 2:1, and 3:1. The colorless area indicates potential emulsions or gel formation, whereas the colored region represents the optimal range for stable nanoemulsion formulation. The phase diagrams indicated that a 1:1 weight ratio of Smix produced the largest nanoemulsion region, making it the preferred choice for nanoemulsion formulation development.

### Nanoemulsion formulations and characterization

**Fig 4:**
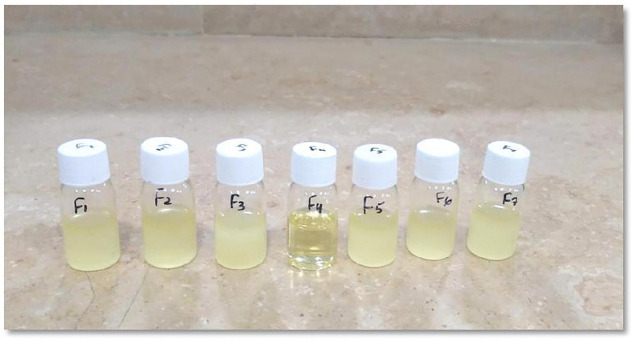
The visual appearance of nanoemulsion formulations (F1-F7).

The compositions of the nanoemulsion formulations (F1–F7) are outlined in Table 2. Fig 4 showed the visual appearance of the developed formulations, which were evaluated for their physicochemical characterization.

**Table 2:** Composition and characterization of *E. intestinalis* extract loaded-nanoemulsions computed by simplex lattice design.

Formulations	Oil (%) X1	Smix (%) X2	Water (%) X3	Droplet size (nm) (Y1)	PDI (Y2)	Viscosity (m-Pa. S) Y3)	Conductivity (µS/cm)	pH	R.I
F1	13.33	73.33	13.33	380 ± 22	0.52	493.33 ± 3.33	12.13 ± 0.19	5.80 ± 0.03	1.443 ± 0.002
F2	15	75	10	390 ± 63	0.47	476.67 ± 3.33	8.43 ± 0.09	5.65 ± 0.01	1.442 ± 0.003
F3	15	65	20	470 ± 65	0.68	696.66 ± 3.33	20.43 ± 0.12	5.72 ± 0.01	1.441 ± 0.001
F4	5	75	20	11.2 ± 12	0.27	170 ± 5.77	21.67 ± 0.09	5.68 ± 0.01	1.43 5 ± 0.002
F5	13.33	68.33	18.33	344 ± 32	0.5	526.67 ± 3.33	18.67 ± 0.09	5.63 ± 0.01	1.444 ± 0.001
F6	8.33	73.33	18.33	183.27 ± 20	0.6	290 ± 5.77	20.83 ± 0.15	5.95 ± 0.003	1.439 ± . 002
F7	11.67	71.67	16.67	304 ± 79	0.57	496.67 ± 3.33	18.30 ± 0.25	5.60 ± 0.01	1.443 ± 0.001

Data are expressed as mean ± SE (n = 3).

According to this study, the designed formulations (F1–F7) exhibited droplet sizes ranging from 11.2 ± 1.2 nm to 470 ± 65 nm, with PDI values varying between 0.27 and 0.68, indicating the hydrodynamic size distribution within the nanoemulsion systems. All formulations (F1–F7) displayed droplet sizes within the nanoemulsion range (below 500 nm), as reported by Sungpud et al. [48]. Moreover, several studies have suggested that the droplet size of topical nanoemulsions falls within the range of 20–500 nm. They provide a greater surface area, long-term stability, enhanced drug absorption, and improved bioavailability [6,15,49]. The degree of globule size uniformity can also be described by PDI values. Notably, larger globule sizes have greater PDI values and vice versa. Lower PDI values suggest a more monodispersed system, which is preferable for sustained effects and uniform release of bioactive metabolites [17]. Despite the PDI values reaching 0.68, all samples remained visually stable. Moreover, all nanoemulsion formulations (F1–F7) displayed PDI values within the acceptable range (0.05–0.7), as reported by Hernández et al. [50].

pH is an important parameter for the characterization of nanoemulsion formulations. Healthy skin maintains a slightly acidic pH, particularly ranging from 4 to 6. An excessively acidic pH can cause skin irritation, whereas a highly alkaline pH (8–9) may promote microbial growth [51]. The pH of nanoemulsions remained within the range of 5.60 ± 0.01 to 5.95 ± 0.003 (Table 2). The pH value falling within the ideal range further corroborates the acceptability of the formulations for topical application [49,52].

The RI values of all nanoemulsions (F1–F7) presented a characteristic of a clear nanoemulsion [53]. All formulations exhibited refractive indices (RI) in the range of 1.435 ± 0.002 to 1.444 ± 0.001 (Table 2). The refractive indices of all formulations fall right above the refractive index of water (1.33) but below that of olive oil (1.465 to 1.468) and most oils. In this study, the continuous phase was likely water, indicating a direct relation with the RI values, which is also mentioned in the previous studies [54,55]. The refractive index value alone may not be sufficient for accurate classification of nanoemulsions as O/W or W/O types. Several factors, such as the specific oil, surfactants, and co-surfactants used, the concentration of each component and the measurement techniques, can influence the overall RI of the emulsion. Techniques such as conductivity measurements and electron microscopy can also be utilized in conjunction with RI measurements to corroborate the emulsion type.

The conductivity measurements reveal the presence of free ions in a formulation and help identify its type. The type and concentration of surfactant can also influence the conductivity of nanoemulsions. W/O emulsions generally have low conductivity (<10 µScm^-1^) due to the low conductivity of oil, whereas O/W emulsions have higher conductivity (>10 µScm^-1^) because of the continuous phase being water [16]. Furthermore, the oil used in the formulation also affects the conductivity [56]. As shown in Table 2, the conductivity values were found to be within the range of 8.43 ± 0.09 to 21.67 ± 0.09. In this study, all nanoemulsion formulations, except F2, were classified as O/W nanoemulsions based on their refractive index and conductivity values. Whereas F2 was classified as a W/O nanoemulsion. These findings are in accordance with the previous study [16].

Viscosity measurement is another important parameter that influences the flow characteristics, spreadability, and stability of the formulation, which will affect the ease of use. Factors such as temperature, pH, composition, and concentration of surfactant and co-surfactant, water, and oil, as well as manufacturing conditions, can influence the viscosity of nanoemulsion formulations [57,58]. The viscosity of the nanoemulsion formulations (F1–F7) ranged from 170 ± 5.77 to 696.66 ± 3.33 m–Pa·S (Table 2), showcasing their suitability for topical applications. The results are aligned with the findings of previous studies [30,59].

### Model fitting and optimization

The Design Expert software generated seven formulations (F1 to F7), detailing the with precise quantities of each ingredient outlined in Table 2. The experimental design analysis revealed a significant impact of the independent variables: oil (X1), Smix (X2), and water (X3) on all measured properties, including droplet size (Y1), PDI (Y2), and viscosity (Y3). In this study, the surfactant-to-co-surfactant ratio varied between 65 and 75%, aligning with previous studies that suggested ratios of approximately 77% [60], and 60% for Tween 80 [24]. Another study also described comparable concentrations of surfactant (77.6–81.8%) and co-surfactant (9.09–11.11%) with olive oil to develop a stable nanoemulsion formulation [61]. To avoid the oily texture of the emulsion, the present study utilized 5–15% (w/w) olive oil, as also described by Hernández et al. [50].

The ANOVA table highlighted the statistical significance of each component of the model. This determines whether the employed model adequately describes the characteristics of the nanoemulsion or not. [57]. According to the present study, the models demonstrated a high correlation with data, with an R^2^ value exceeding 0.90 (Table 3). In this study, high R^2^ values were achieved, suggesting that the model was highly efficient in fitting the data under experimental conditions. The adjusted R^2^ values indicated reasonable agreement between the predicted and experimental values of the model for all measured responses. The difference between adjusted and predicted R^2^ values was not more than 0.2, indicating that the model was well-fit to the data [30]. Adequate precision measures the signal-to-noise ratio, a ratio greater than 4, proposing that the model was acceptable for all responses. Table 3 indicates an adequate signal strength for all response variables, and the model can be used to navigate the design space. Additionally, the low Coefficient of Variation (C.V. %) suggests precise measurements, while the PRESS (Predicted Residual Error Sum of Squares) values for PDI, droplet size, and viscosity demonstrated the good predictive capabilities of the model. Moreover, the *p-*values <0.05 corroborate the significance of the model fitting according to ANOVA. The model summary is presented in Table 3.

**Table 3:** Summary of the model statistics.

Variables	R^2^	Adjusted R^2^	Predicted R^2^	Adeq. Precision	Mean	SD	Press	% CV	P-value
Droplet size	0.9999	0.9998	0.9892	260.218	297.496	1.904	1531.88	0.640	0.0086
PDI	0.9996	0.9974	0.8151	67.0122	0.5164	0.0065	0.018	1.26	0.035
Viscosity	0.9707	0.956	0.9291	21.9674	450	35.88	12447.78	7.97	0.0009
Remarks	Significant interaction of Excipients								

(SD), Standard deviation; (PDI), Polydispersity index; (PRESS), Predicted residual error sum of squares.

**Fig 5:**
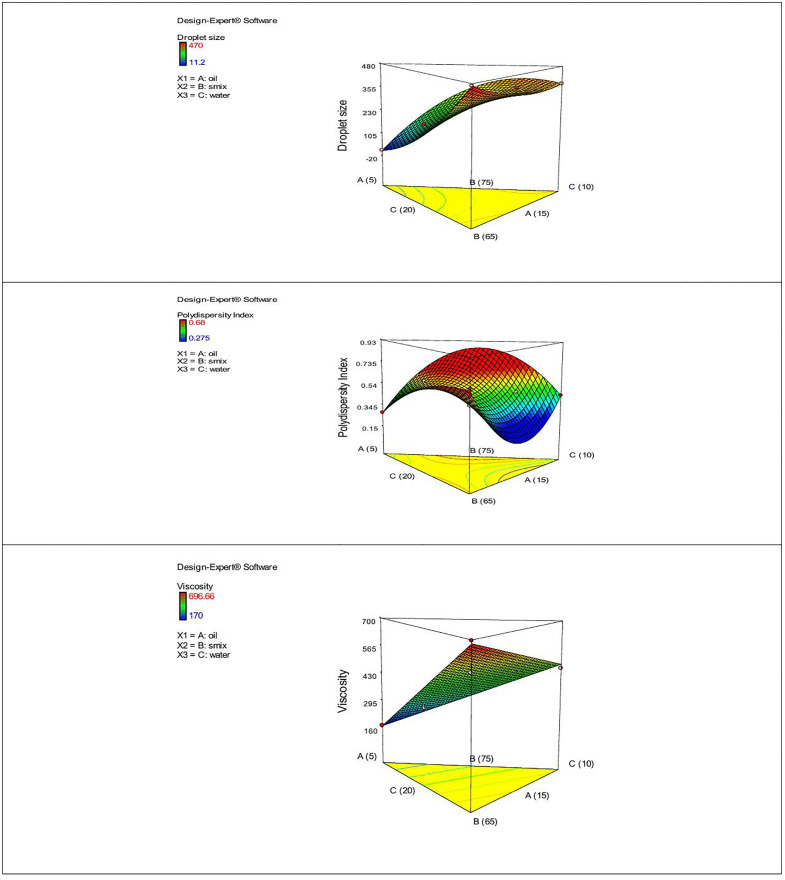
3D surface plots demonstrating the effect of the independent variables; oil (A), Smix (B), and water (C) and dependent variables; globule size (nm), PDI, and Viscosity.


The ANOVA analysis indicated that oil and Smix concentrations significantly influenced droplet size, with a P-value of 0.0086. An increase in oil content generally led to larger droplets, whereas a higher Smix concentration results in smaller globules, as observed in the F4 formulation (droplet size 11.2 nm). Notably, formulation with the lowest Smix concentration (F3) produced larger droplets (470 ± 65 nm). This may be due to the localization of surfactant molecules at the oil-water interface, which stabilizes the interfacial film and oil droplets. This effect is widely observed in previous studies [22,62,63]. Additionally, the water content also affects the droplet size. Water in combination with surfactant also reduced the droplet size, as previously described [30]. The 3D plot (Fig 5) and the below-mentioned polynomial equation summarized these correlations as follows:

Droplet size =89.970A+35.773B+17.293C−2.885AB+6.235AC−2.0062BC (4)

The quadratic model displayed a smaller PDI value with increasing oil content, potentially due to the stabilizing effect of olive oil [63]. The mutual effect of water and Smix presented an antagonistic effect, as F4 exhibited the lowest PDI with a value of 0.27. While increasing one of them (Smix or water proportion) resulted higher PDI. Notably, the F3 formulation, which had the lowest surfactant with high water content, displayed a higher PDI (0.68). This may lead to formulation instability and globule coalescence. The presented data were comparable to the findings of an earlier work [30]. The results showed a significant effect on the response variable, with a p-value of 0.035, which substantiates the fitness of this model.


PDI= −0.853977A+0.120341B+0.823091C+0.004814AB+0.019814AC−0.016486BC (5)

According to equation and the 3D plot (Fig 5), the viscosity exhibited a direct and indirect relation of oil and Smix concentration, as seen in all formulations (F1 to F7). The F4 formulation, which had a lower oil content (5%) and higher Smix content, exhibited low viscosity (170 ± 5.77 m-Pas. S). While F3 displayed higher viscosity (696.66 ± 3.33 m-Pas.S) due to its low Smix concentration and increased oil content. F2, despite having the greatest Smix concentration, exhibited higher viscosity (476.67 ± 3.33 m. Pas-S), which may be due to the higher oil content (Table 2). A higher viscosity might result from the oil, which may hinder the Smix’s capability to emulsify the oil globules effectively. Yousry et al. [30] described a similar trend of the oil effect on viscosity. The results also revealed that a reduced amount of Smix increased the viscosity, as it may raise the interfacial tension between water and oil, producing a more viscous emulsion. The results were consistent with those of a previous study described by Che Marzuki et al. [58]. These findings suggested that the combined effect of oil and Smix content determines the overall viscosity of the formulations. Furthermore, the ANOVA results revealed that the data fit a linear model with a statistically significant p-value (0.0009).


Viscosity = 46.92372A−4.67548B+14.25772C (6)

Overall, these findings highlighted a significant and predictable interaction between excipients in the formulation as well as provided the conditions for optimizing the nanoemulsion formulations. The positive and negative signs in the aforementioned equations denote the synergistic and antagonistic effects, respectively.

Droplet size is the foremost influencing parameter for the stability of nanoemulsions and the effective transport of active ingredients to the skin [50]. Accordingly, the designed nanoemulsion formulation was optimized considering droplet size as a major parameter along with acceptable PDI and desirable viscosity for topical application. Therefore, among the studied formulations (F1–F7), F6 was chosen as the optimal formulation. Due to its droplet size of 183.27 ± 20.04 nm, which closely aligns with the ideal droplet size range (20–200 nm) and PDI value (0.6), falling within the acceptable range (0.05–0.7) as described previously [18,50]. Although formulation F4 achieved the smallest droplet size and PDI value, it was not selected because it contained the lowest amount of extract and oil and higher surfactant levels, which may accelerate droplet coalescence and rapid destabilization. Furthermore, droplet sizes below 20 nm are prone to being rapidly cleared from the skin surface [39] or can diffuse into the viable epidermis without penetrating the skin [64], thus limiting their therapeutic efficacy. An excessively high viscosity might hinder the formulation process, while a lower viscosity would affect the stability [30]. The chosen formulation (F6) showed optimal viscosity (290 ± 5.77m-Pa.S) and was further evaluated for stability and biological investigation.

### Stability studies

The selected nanoemulsion formulation (F6) was stable at 4 ± 2 ^°^C and 25 ± 2 ^°^C throughout the storage period. Any signs of phase separation, creaming, precipitation, or color changes were not recorded, though a slight decrease in pH was noted while being stored at 25 **±** 2 ^°^C, but stable at 4 ± 2 ^°^C (Table 4).

**Table 4:** Stability studies of nanoemulsion formulation (F6) at different storage temperature.

Parameters	At fresh state		After 1 month			After 2 month			After 3 month		
	Temperature										
	4 ± 2 ^°^C	25 ± 2 ^°^C		4 ± 2 ^°^C	25 ± 2 ^°^C		4 ± 2 ^°^C	25 ± 2 ^°^C		4 ± 2 ^°^C	25 ± 2 ^°^C
**Color**	Y	Y		Y	Y		Y	Y		Y	Y
**Clarity**	C	C		C	C		C	C		C	C
**Texture**	NG	NG		NG	NG		NG	NG		NG	NG
**Phase separation**	–	–		–	–		–	–		–	–
**pH**	5.95 ± 0.003	5.95 ± 0.003		5.94 ± 0.029	6.03 ± 0.024		5.92 ± 0.069	5.78 ± 0.050		5.64 ± 0.085	5.49 ± 0.058

(Y), Yellow; (C), Clear; (NG), non-greasy; *“*-*”* means no phase separation. (pH values: mean ± SEM).

**Fig 6:**
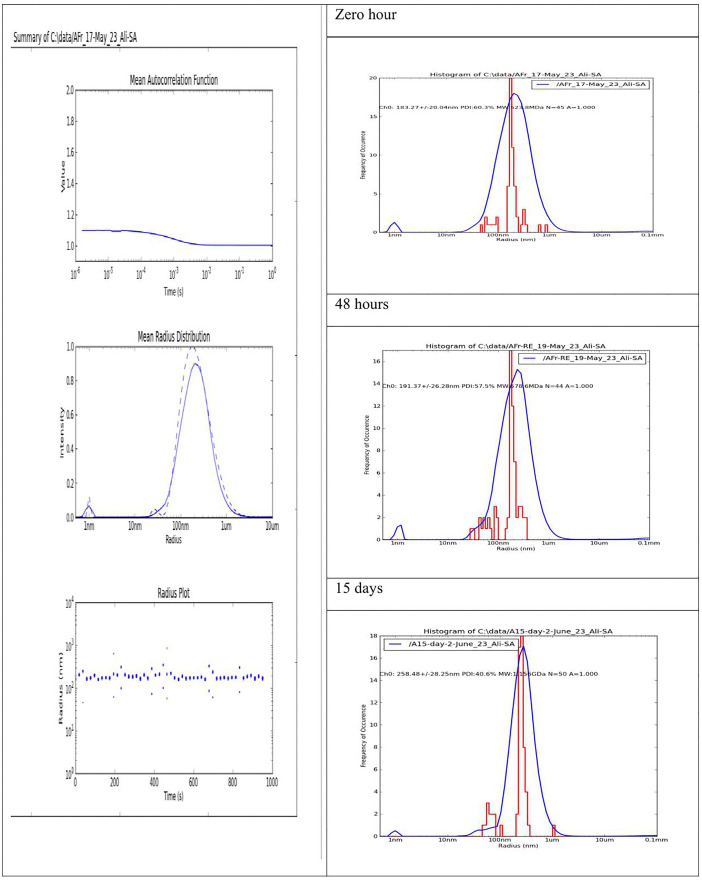
Measurement of the Droplet size of *E. intestinalis* based-NE at various time intervals. (Left panel) DLS results of NE illustrating the experimental conditions, i.e., the mean autocorrelation function, monodispersity and radius, respectively. (Right panel) Comparative corresponding droplet size of NF at various time intervals (from top to bottom panel, respectively). All experiments were performed with an auto-piloted run of 50 measurements at every 20 s, with a wait time between of 1 s (at 25 ^°^C).

Furthermore, the droplet size varied from 183.27 to 300.25 ± 63 nm, yet it consistently fell within the nanoemulsion range. Interestingly, the PDI value did not increase; instead, a slight decrease was noted, which was likely due to the stabilizing effect of olive oil [63]. The PDI value remained within the acceptable range of 0.40 to 0.60, as noted at 0 (fresh state), 3, 15, and 90 days of storage (Table 5 and Fig 6). The findings of stability studies are consistent with those of previous studies [50,59], suggesting that storage at 4 ^°^C yields optimal results.

**Table 5:** Droplet size and PDI of F6 nanoemulsion formulation at various time intervals.

Variables	Time intervals			
	1^st^ day/Fresh	3^rd^ day	15 day	90 day
Droplet size	183.27 ± 20.04	191.37 ± 26.28	258.48 ± 28.25	269.9–300.25 ± 63
PDI	0.60	0.57	0.40	0.52

### Extract-excipients interaction studies

**Fig 7:**
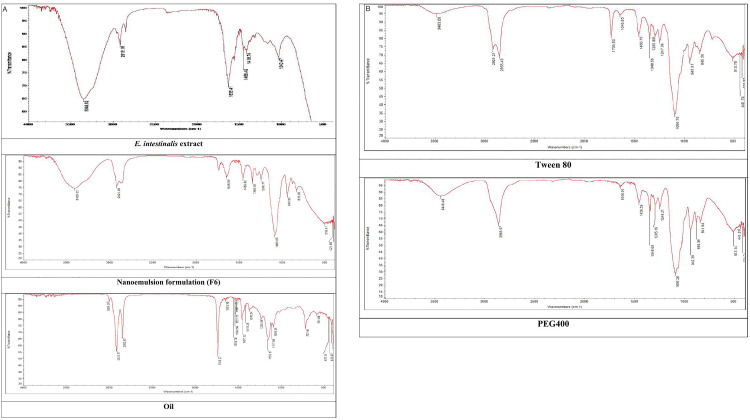
continued.

FT-IR is a powerful analytical tool that is extensively employed to study the potential interactions between the drug and excipients used in the formulation. FT-IR characterization provides detailed information on the specific functional groups in bioactive compounds [17]. The FT-IR spectrum presented in Fig 7A revealed multiple absorption bands, suggesting the occurrence of various functional groups in *E. intestinalis* extract and its corresponding nanoemulsion (F6). The absorption at 3349.62 cm^-1^ represents O-H stretching vibrations, suggesting the presence of polysaccharides, flavonoids, and terpenes [65,66]. While the peak 2916.16 cm^-1^ is related to the C-H stretching showed alkane. Moreover, the C = O stretching appeared at 1635.41 cm^-1^ showed the vibration in proteins or the N-H bond in the primary amine. The absorption at 1456.49 cm^-1^ indicates COO-vibration stretching [67], whereas the peak at 1037.7 cm^−1^ is associated with C-O stretching, indicating the presence of the flavonoid ring [68]. Notably, the FT-IR spectrum showed similar functional groups in the extract and extract-loaded nanoemulsion (F6), with slight shifts in the peak positions of O-H, C-H, COO, and C-O stretching vibrations towards higher wavelengths (Fig 7B). These findings indicated physisorption, in which the extract molecules stick to the nanoemulsion surface through weak Van der Waals forces, and did not substantially alter the chemical structural integrity of the extract components [65].

### Spreadability

For topical applications, optimal spreadability is essential to achieve the desired effect as well as to enhance user compliance [69]. An excessively high spreadability value can lead to product wash-off, while a low value can hinder proper application [70]. The F6 nanoemulsion showed a good spreadability, with a value of 34.28 ± 1.81 g.cm/s, that demonstrating their suitability for smooth application, comparable to the value reported in an earlier study [71].

### Antioxidant activity

*E. intestinalis* is a substantial source of various bioactive molecules with antioxidant potential. These compounds neutralize free radicals, thereby preventing oxidative damage [12]. Accordingly, this study utilized *E. intestinalis* extract to design a nanoemulsion formulation, and its antioxidant and anti-inflammatory properties were evaluated. The results demonstrated that encapsulating the EI extract within a nanoemulsion formulation significantly (*p* < 0.05) enhanced its antioxidant activity by both methods: DPPH radical scavenging and FRAP assays. DPPH is a simple and reliable method for measuring a molecule’s radical scavenging ability, resulting in a distinct color change [72]. The EI extract and nanoemulsion displayed percentage inhibition ranged from 44.03 ± 0.03 to 50.57 ± 0.05% and 48.75 ± 0.06 to 54.32 ± 0.19%, respectively, at equivalent concentration (50–800 µg/mL). The IC_50_ values for the extract and extract-loaded nanoemulsion were 660.85 μg/mL and 163.19 μg/mL, respectively. A lower IC_50_ value was noted for the nanoemulsion formulation compared to the extract alone. Notably, the IC_50_ value of ascorbic acid exhibited the lowest IC_50_ value of 4.768 µg/mL (Table 6).

**Table 6:** Antioxidant activity of *E. intestinalis* extract and F6 nanoemulsion.

% Inhibition by DPPH assay					% Reduction by FRAP assay		
Concentration (μg/mL)	Ascorbic acid	Concentration (μg/mL)	*E. intestinalis* extract	EI-NE	Concentration (μg/mL)	*E. intestinalis* extract	EI-NE
5	45.41 ± 0.11	50	44.03 ± 0.03	48.75 ± 0.06^*^	200	10.53 ± 1.22	40.91 ± 0.09^*^
10	54.22 ± 0.14	100	46.86 ± 0.03	49.68 ± 0.07^*^	400	16.14 ± 0.93	51.16 ± 0.12^*^
20	87.01 ± 0.09	200	47.08 ± 0.09	50.31 ± 0.06^*^	800	32.98 ± 0.35	61.75 ± 0.15^*^
40	97.44 ± 0.06	400	48.73 ± 0.03	52.23 ± 0.06^*^	1600	43.51 ± 1.26	71.79 ± 0.95^*^
–	–	800	50.57 ± 0.05	54.32 ± 0.19^*^	3200	51.58 ± 0.61	88.42 ± 0.61^*^
IC_50_	4.768 μg/ml	–	660.85 μg/ml	163.19 μg/ml	–	–	–

The values represented as means ± SD. *p < 0.05 using paired t-test.

The FRAP assay is based on the reduction of Fe^+++^ to Fe^++^ ions in a potassium ferricyanide (III) [K_3_Fe(CN)_6_] solution, which forms a Prussian blue-colored complex [72,73]. The extract showed a reducing power ranging from 10.53 ± 1.22 to 51.58 ± 0.61%, while the extract-based NE displayed 40.91 ± 0.09 to 88.42 ± 0.61%, respectively. The results are depicted in Table 6. The reduction observed with the *E. intestinalis* extract was aligned with other species of Enteromorpha, such as *E. compressa* and *E. Prolifera* [34]. Both the extract and algal-based nanoemulsion demonstrated concentration-dependent inhibition and reducing power, with the nanoemulsion displaying superior activity (*p* < 0.05). Pathania et al. and Gomathy et al. [23,74] also stated that the incorporation of nanoparticles and nanoemulsions significantly enhances the antioxidant potential of crude extracts. The metabolites, including phenolics (phenanthrene, cinnamic acid, ellagic acid, and quinic acid) and flavonoids (apigenin, rutin, diosmin, and quercetin), present in the *E. intestinalis* extract are most likely attributed to this enhanced antioxidant activity [12]. Both techniques demonstrated extended antioxidant activity (*p* ≤ 0.05) of *E. intestinalis-*based nanoemulsion (F6).

### Paw edema model

**Fig 8:**
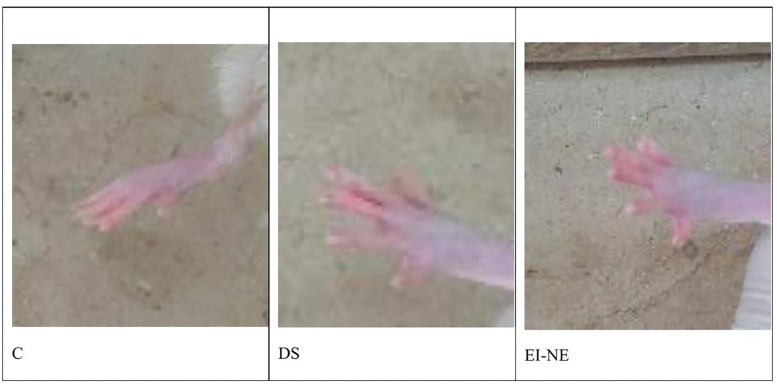
Anti-inflammatory effect of nanoemulsion formulation F6 on Paw edema (C; Control, DS; Diclofenac sodium, EI-NE; *E. intestinalis* nanoemulsion).

The carrageenan-induced paw edema model is extensively employed to assess the efficacy of anti-inflammatory drugs in experimental animals. The *E. intestinalis*-based nanoemulsion (F6) displayed significant anti-inflammatory activity (*p* ≤ 0.05), with edema reduction ranging from 8 to 31%, while the standard drug (diclofenac sodium) displayed 12–41% inhibition. The F6 nanoemulsion formulation presented a maximum inhibition of 30%, whereas the standard drug (diclofenac sodium) achieved a maximum inhibition of 40% at 4 h. The results are illustrated in Table 7 and Fig 8. One-way ANOVA followed by Dunnett’s post hoc analysis showed that both the standard drug and algal-based nanoemulsion notably reduced paw edema volume in the early and late phases of inflammation compared to the control group (*p* ≤ 0.05), with some effects sustained for up to 4 hours after topical application. These results are aligned with those of previous research [37,75]. Furthermore, *E. intestinalis* has the potential to reduce inflammation due to the presence of bioactive substances, such as phenolics and fatty acids, as identified through GC-MS and FT-IR spectrophotometric analysis. These metabolites are known for their ability to counteract oxidative stress and inflammation, as highlighted in earlier studies [12,19]. The nanoemulsion formulation serves as an effective carrier for crude extracts by incorporating phytonutrients, including vitamins, antioxidants, and fatty acids, all of which play vital roles in combating oxidative stress and related inflammatory conditions. The inclusion of olive oil in the nanoemulsion further contributed to its inherent anti-inflammatory properties.

**Table 7:** Anti-inflammatory effect of F6 nanoemulsion on Paw edema.

Treatments	% inhibition of Paw edema volume					
	0 min	30 min	1 h	2h	3h	4h
Control	0.76 ± 0.01	1.05 ± 0.02	1.62 ± 0.04	2.33 ± 0.05	3.54 ± 0.03	3.75 ± 0.02
EI-NE	0.75 ± 0.01	0.96 ± 0.00^**^ (8%)	1.36 ± 0.01^**^ (16%)	1.79 ± 0.01^**^ (23%)	2.46 ± 0.02^**^ (31%)	2.61 ± 0.02^**^ (30%)
Diclofenac sodium	0.77 ± 0.1	0.92 ± 0.01^**^ (12%)	1.19 ± 0.001^**^ (27%)	1.56 ± 0.04^**^ (33%)	2.10 ± 0.03^**^ (41%)	2.24 ± 0.03^**^ (40%)

Values are expressed as Mean ± SEM (n = 6). ^**^P < 0.05 compared to the control group. Difference between groups were analysed by one-way analysis of variance (ANOVA) followed by Dunnett’s post hoc test.

According to these findings, the F6 nanoemulsion formulation displayed an anti-inflammatory effect comparable to that of commercially available diclofenac sodium gel. The anti-inflammatory activity results from greater permeability and solubility of bioactive compounds, which are encapsulated within the nanoemulsion system, thereby producing a sustained biological effect. Therefore, it can be used for the management of various inflammatory diseases.

### Acute dermal toxicity

**Fig 9:**
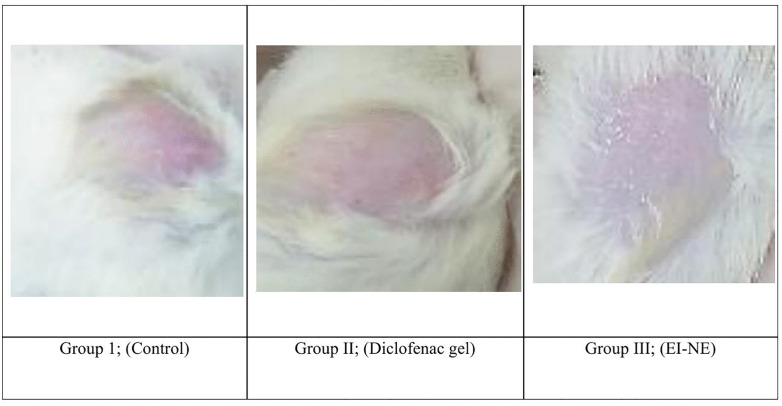
Acute dermal toxicity assessment on rats’ skin.

Skin irritation was assessed using the Draize dermal irritation scoring scale, ranging from 0 (no irritation) to 4 (severe irritation) based on redness (erythema) and swelling (edema). Fig 9 illustrates that the EI-NE formulation didn’t show any signs of erythema or edema following 24 hours of treatment. The formulation was found to be non-irritating, with a PII score of 0.00. While diclofenac gel induced mild redness in one animal, swelling was not recorded (PII value: 0.17 ± 0.1). The results indicate the safety profile of the nanoemulsion formulation.

## Conclusions

This study represents the first documented attempt to develop an *E. intestinalis* extract-based nanoemulsion with antioxidant and anti-inflammatory effects. Among the seven formulations (F1–F7), F6 was designated as the optimal formulation based on the model-predicted optimization criteria, which included an appropriate droplet size within the range of 20–200 nm, acceptable PDI, and optimal viscosity for topical application. The formulation O/W nanoemulsion was clear, non-toxic, stable, and had appropriate spreadability, ensuring optimal skin contact for the desired effects, making it useful for topical applications. Additionally, the antioxidant and anti-inflammatory activities of the formulation show promise for potential applications in both pharmaceuticals and cosmeceuticals. These findings not only validate the effectiveness of the designed nanoemulsion formulation for topical applications but also lay the groundwork for future innovations. This advancement paves the way for further *in vivo* studies to evaluate its efficacy and safety for clinical applications.

## Supporting information

S1 Table GC-MS profile of *E. intestinalis* extract. (XLSX)

S2 SPSS Data Scavenging activity of *E. intestinalis* extract and nanoemulsion. (XLSX)

S3 SPSS Data Ferric reducing power assay of *E. intestinalis* extract and nanoemulsion. (XLSX)
